# Transcriptome-Wide Analysis Reveals the Role of PPAR*γ* Controlling the Lipid Metabolism in Goat Mammary Epithelial Cells

**DOI:** 10.1155/2016/9195680

**Published:** 2016-10-13

**Authors:** Hengbo Shi, Wangsheng Zhao, Changhui Zhang, Khuram Shahzad, Jun Luo, Juan J. Loor

**Affiliations:** ^1^College of Life Science, Zhejiang Sci-Tech University, Hangzhou, Zhejiang 310018, China; ^2^Zhejiang Provincial Key Laboratory of Silkworm Bioreactor and Biomedicine, Hangzhou, Zhejiang 310018, China; ^3^Shaanxi Key Laboratory of Molecular Biology for Agriculture, College of Animal Science and Technology, Northwest A&F University, Yangling, Shaanxi 712100, China; ^4^School of Life Science and Engineering, Southwest University of Science and Technology, Mianyang 621010, China; ^5^Mammalian NutriPhysioGenomics, Department of Animal Sciences and Division of Nutritional Sciences, University of Illinois, Urbana, IL 61801, USA

## Abstract

To explore the large-scale effect of peroxisome proliferator-activated receptor *γ* (*PPARG*) in goat mammary epithelial cells (GMEC), an oligonucleotide microarray platform was used for transcriptome profiling in cells overexpressing* PPARG* and incubated with or without rosiglitazone (ROSI, a PPAR*γ* agonist). A total of 1143 differentially expressed genes (DEG) due to treatment were detected. The Dynamic Impact Approach (DIA) analysis uncovered the most impacted and induced pathways “fatty acid elongation in mitochondria,” “glycosaminoglycan biosynthesis-keratan sulfate,” and “pentose phosphate pathway.” The data highlights the central role of* PPARG* in milk fatty acid metabolism via controlling fatty acid elongation, biosynthesis of unsaturated fatty acid, lipid formation, and lipid secretion; furthermore, its role related to carbohydrate metabolism promotes the production of intermediates required for milk fat synthesis. Analysis of upstream regulators indicated that* PPARG* participates in multiple physiological processes via controlling or cross talking with other key transcription factors such as* PPARD* and* NR1H3* (also known as liver-X-receptor-*α*). This transcriptome-wide analysis represents the first attempt to better understand the biological relevance of PPARG expression in ruminant mammary cells. Overall, the data underscored the importance of PPARG in mammary lipid metabolism and transcription factor control.

## 1. Introduction

Ruminant milk products are now common and popular throughout the world. Milk fat is an important component of dairy products and is a major contributor to dietary energy density. The higher concentrations of unsaturated and medium-chain fatty acids are responsible for the characteristic “goaty” odour of goat milk and also confer unique organoleptic properties [1]. Therefore, understanding the mechanisms for altering the milk fatty acid composition of goat milk may lead to further improvements in nutritional value. Recent evidence indicates that milk fat biosynthesis is regulated by key transcription factors including peroxisome proliferator-activated receptor *γ* (*PPARG*) [2, 3].

It is well established that* PPARG* is a critical transcription factor controlling adipogenesis and glucose metabolism in various cells in nonruminants [4–6]. After binding of ligands (e.g., rosiglitazone (ROSI) or pioglitazone),* PPARG* causes conformational changes in the receptor [7, 8] and then forms a heterodimeric complex with RXR proteins and binds to PPAR response element (PPRE) upstream of target genes [9]. Through controlling the downstream genes,* PPARG* regulates adipocyte differentiation and promotes insulin sensitivity in human and rodents [7]. The activation of PPARG also enhances macrophage lipid uptake as well as lipid export and has anti-inflammatory effects [10].

In bovine cells, the activation of* PPARG* with rosiglitazone provided a demonstration that PPARG could control expression of genes involved in milk fat synthesis [11]. The current data from goats indicates that* PPARG* regulates genes involved in triacylglycerol synthesis and secretion in mammary gland epithelial cells [12]. It was also demonstrated that* PPARG* stimulates the synthesis of monounsaturated fatty acids in dairy goat mammary epithelial cells (GMEC) via the control of stearoyl-coenzyme A desaturase (*SCD*) [2]. Furthermore, our recent data revealed that PPARG could modulate lipid accumulation via regulation of Perilipin 2 (*PLIN2*) gene expression in GMEC [13]. Although some work [2, 3] has been performed to study the function of* PPARG* in ruminant mammary cells, a comprehensive dataset on gene profiles altered by* PPARG* is not available.

Microarray analysis provides an efficient tool to simultaneously study the expression of multiple genes in tissues or cells in response to a given treatment or physiological condition. It has been widely used in the bovine to study the differential gene expression among different treatments or physiological conditions [14–16]. Structural genomic studies of domestic animals have indicated that goats are closely related to bovine species [17]. Previous evidences were highly suggestive that cross-species hybridization is possible using a bovine cDNA microarray to study goat gene expression [18–20].

The primary aim of this study was to assess the potential role of* PPARG* in GMEC at global scale. To that aim, a microarray analysis was used to detect the transcriptome alterations of GMEC after overexpression of* PPARG*. The results indicated that* PPARG* gain of function induced more than 1,000 differentially expressed genes (DEG), most of which are related to metabolism pathways.

## 2. Experimental Section

### 2.1. Cell Culture and Treatments

The mammary epithelial cells were isolated from peak lactation Xinong Saanen goats as described previously [21]. Details of cell culture were described recently [3, 12]. Cultures of GMEC at approximately 80% confluence were transfected with one of the adenovirus supernatants (Ad-PPARG or Ad-GFP). Transfected GMEC were cultured with the PPARG-specific ligand ROSI (BioVision, USA) (PPARG+ROSI) or control [dimethyl sulfoxide (DMSO)] (Sigma, St. Louis, MO, USA) (PPARG+DMSO and Ad-GFP+DMSO) at 50 *μ*M after 24 h of the initial culture and then harvested at 48 h (24 h later) for RNA extraction. The generation and application of the adenovirus expression PPARG (Ad-PPARG) were described elsewhere [2]. Each treatment was performed in triplicate.

### 2.2. Total RNA Extraction

The procedures for total RNA extraction, purification, and qPCR were recently described [22]. Total RNA from GMEC was extracted using the RNA Prep pure cell kit (Tiangen Biotech Co. Ltd., Beijing, China) according to the manufacturer's protocol. The RNA used in the qPCR was treated with DNAase (Tiangen Biotech Co. Ltd., Beijing, China) to remove genomic DNA contamination. Synthesis of cDNA was conducted using the Prime Script*™* RT kit (Takara Bio Inc., Otsu, Japan) according to the manufacturer's instructions.

### 2.3. Microarray

An Agilent platform was chosen to conduct the microarray experiment (44K Bovine (V2) gene expression microarray chip, Agilent Technologies Inc.) following the manufacturer's protocols. Briefly, a total of 200 ng of RNA per sample were used to generate first-strand cDNA, which was reverse transcribed to cRNA using the low-input quick amp labeling kit (Agilent Technologies Inc.). The resulting cRNA was labeled with either Cy3 or Cy5 fluorescent dye, purified using RNeasy minispin columns (Qiagen), and subsequently eluted in 30 *μ*L of DNase-RNase-free water. The NanoDrop ND-1000 (Thermo Fisher Scientific Inc., Waltham, MA) and a Bioanalyzer 2100 (Agilent Technologies) were used to confirm the manufacturer's recommended criteria for yield of at least 0.825 *μ*g/*μ*L and RNA integrity ≥6, respectively.

### 2.4. Quantitative Real-Time PCR (qPCR)

The results from microarray were validated via qPCR for a selected panel of 15 genes considered important for fatty acid metabolism. The gene names and primers used in this study are reported in Supporting File 1 (in Supplementary Material available online at http://dx.doi.org/10.1155/2016/9195680). Methods for primer pair design and validation and qPCR were as previously described [12]. Data of qPCR were normalized to three internal control genes, Ubiquitously Expressed, Prefoldin-Like Chaperone (*UXT*), Mitochondrial Ribosomal Protein L39 (*MRPL39*), and Ribosomal Protein S9 (*RPS9*).

### 2.5. Data Analysis

Data from microarrays were normalized using Lowess prior to statistical analysis using ANOVA in GeneSpring (Agilent Technologies). Differences in relative expression between PPARG versus CON, PPARG+ROSI versus CON, and PPARG+ROSI versus PPARG were considered significant at an unadjusted *P* < 0.05 and a fold change greater or lower than 2 [23]. The qPCR data were log⁡_2_ transformed prior to statistical analysis. The data were analyzed using a Generalized Linear Model (GLM) using SAS with treatments (CON, PPARG, and PPARG+ROSI) as the main effect. Significance was declared at *P* < 0.05.

### 2.6. Data Mining

Data were mined by an integrative systems biology approach applying the newly developed Dynamic Impact Approach (DIA) [24] and an upstream gene network analysis using Ingenuity Pathway Analysis (IPA) [14]. The Kyoto Encyclopedia of Genes and Genomes (KEGG) pathways and Gene Ontology (GO) biological process category database of bovine were used for functional analysis with the DIA. The detailed methodology for data analysis using DIA was described previously [14]. The IPA Knowledgebase is used to predict the expected causal effects between upstream regulators and targets (i.e., DEG).

## 3. Results

### 3.1. Number of Differentially Expressed Genes (DEG) in the Microarray Data

**Figure 1:**
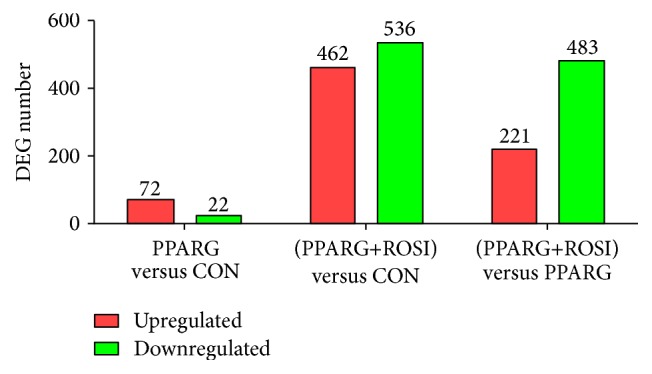
Differentially expressed genes in goat mammary epithelial cells across different treatment comparisons. Cells overexpression of peroxisome proliferator-activated receptor-*γ* (PPARG) with rosiglitazone (ROSI) (PPARG+ROSI) versus CON (cells treated with adenovirus expressing GFP), PPARG versus CON, and PPARG+ROSI versus PPARG.

Overall, there were more than 1,398 DEG detected by microarray. Among these, only the genes (1143) annotated with a bovine Entrez gene ID with a significant difference (*P* < 0.05) and 2-fold change ratio were used for the analysis. The number of DEG indicated a marked difference in expression in the cells overexpressing PPARG with ROSI compared with cells without ROSI (Figure 1). Compared with control, there were 464 DEG upregulated and 536 DEG downregulated in PPARG+ROSI versus CON. The overexpression of PPARG alone did not markedly alter the transcriptome, but there were 72 upregulated and 22 downregulated genes. When compared with cells expressing PPARG with and without ROSI, the analysis indicated that the number of upregulated and downregulated DEG was 221 and 483, respectively.

### 3.2. Overall Summary of KEGG Categories

Using the DIA, the estimate of the perturbation in a biological pathway is represented by the “impact” while the overall direction of the perturbation is represented by the “flux” (or Direction of the Impact) [24]. The DIA provides a summary of the KEGG pathways in the form of categories and subcategories (Figure 3) which are altered by treatments. The details of each pathway are reported in Supporting File S3.

**Figure 2:**
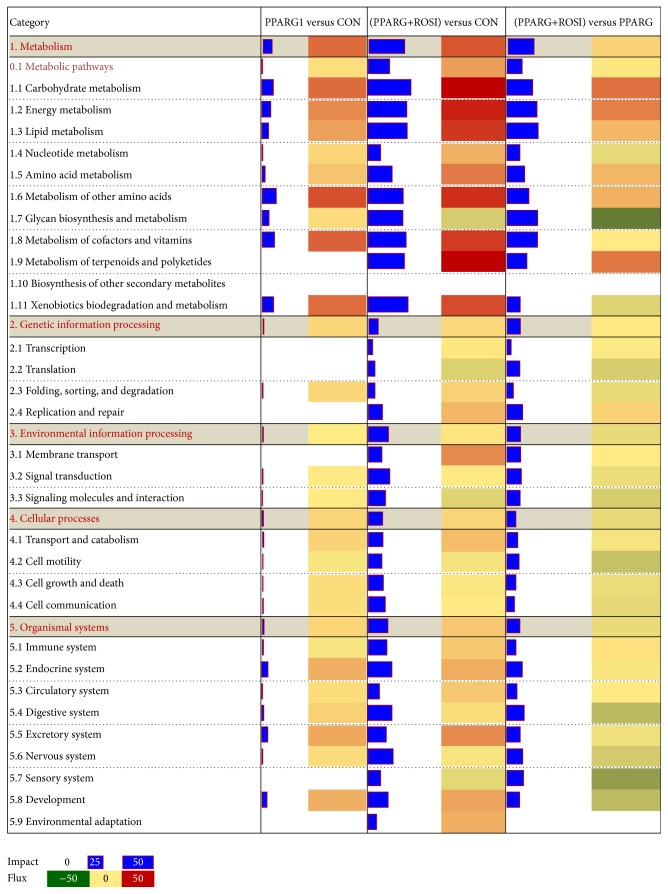
The summary of KEGG pathways provided by the Dynamic Impact Approach (DIA). The “impact” is represented by the horizontal blue bars (the larger the bar, the larger the impact) and the “flux” (Direction of the Impact) is represented by green (more inhibited) to red (more activated) rectangles.

In accordance with the number of DEG in Figure 1, KEGG pathway categories were more impacted in the two comparisons related to cells treated with ROSI. Among these pathways, the category “metabolism” was the most impacted (Figure 2). With the exception of the subcategories of pathways within “biosynthesis of other second metabolites,” “nucleotide metabolism,” and “amino acid metabolism,” all the other subcategories within metabolism had an impact value >25 in the comparison of PPARG+ROSI with CON.

A similar induction effect was uncovered in the comparison of PPARG+ROSI with PPARG. Except for the minor inhibition of “glycan biosynthesis and metabolism,” in the comparison of PPARG+ROSI with CON, most of the metabolic pathways were markedly activated including “carbohydrate metabolism,” “energy metabolism,” “lipid metabolism,” “amino acid metabolism,” “metabolism of other amino acids,” “glycan biosynthesis and metabolism,” “metabolism of cofactors and vitamins,” “metabolism of terpenoids and polyketides,” and “xenobiotics biodegradation and metabolism.” Compared with the control group, only the overexpression of PPARG had a weaker impact on pathway categories except “metabolism.”

According to the impact value, the categories “genetic information processing,” “environment information processing,” “cellular process,” and “organismal system” also were altered in the comparison of PPARG+ROSI with CON. However, most of their flux values were slightly activated or did not change. In PPARG+ROSI versus PPARG, the fluxes in the four categories were inhibited or exhibited no change.

### 3.3. Most Impacted KEGG Pathways

**Figure 3:**
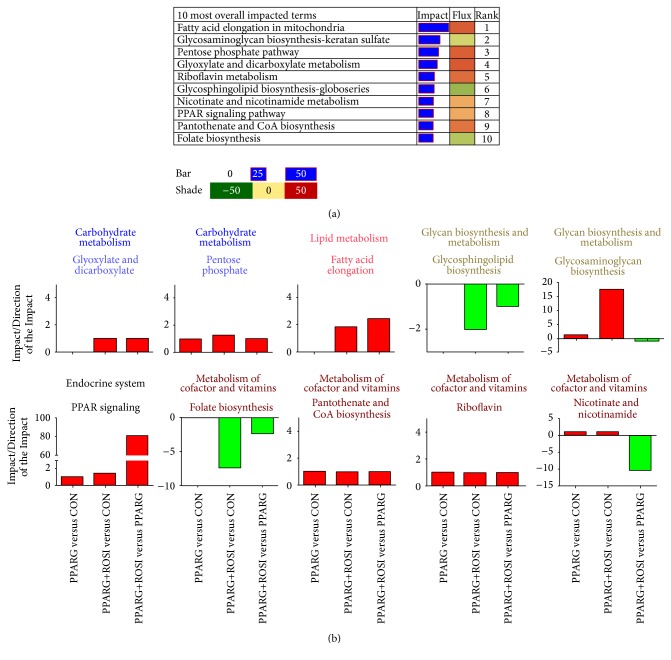
Dynamic Impact Approach (DIA) results for the 10 most impacted KEGG pathways. (a) The overall 10 most impacted pathways and rank. (b) The impact/Direction of the Impact of 10 most impacted pathways in each comparison.

The DIA analysis revealed that the most impacted pathway was “fatty acid elongation in mitochondria” with flux >60, followed by “glycosaminoglycan biosynthesis” (Figure 3). The categories containing “fatty acid elongation in mitochondria,” “pentose phosphate pathway,” “glyoxylate and dicarboxylate metabolism,” “riboflavin metabolism,” “nicotinate and nicotinamide metabolism,” “PPAR signaling pathway,” and “pantothenate and CoA biosynthesis” were highly activated. In contrast, the pathways “glycosphingolipid biosynthesis-globoseries” and “folate biosynthesis” were inhibited.

Even though “glycosaminoglycan biosynthesis” was the second most impacted pathway, it was slightly inhibited by the activation of* PPARG*. “Glycosphingolipid biosynthesis” was highly inhibited with the activation of PPARG. Among the top ten overall most impacted terms, only PPARG belonged to “endocrine system”; the rest of them belonged to “metabolism” (Figure 3).

### 3.4. Expression of Selected Genes by qPCR

**Figure 4:**
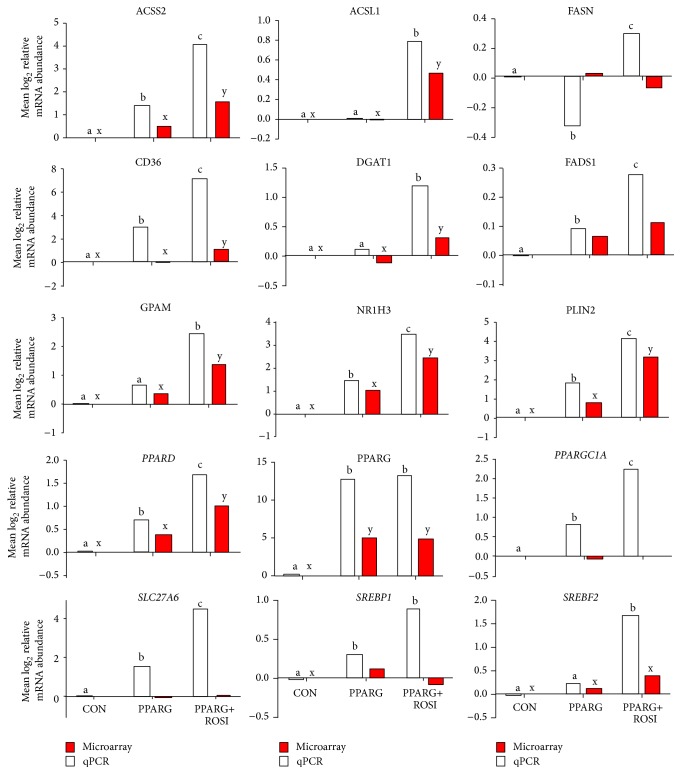
qPCR analysis of selected transcripts and comparison with microarray data. Several of selected transcripts were present and differentially expressed among the comparison in the microarray data and qPCR. a, b, and c denote differences with *P* < 0.05 in qPCR data and x and y denote differences with *P* < 0.05 in microarray data.

Fifteen genes considered important for fatty acid metabolism were selected to assess the reliability of the microarray data. Overall, >80% of genes measured by qPCR had a result deemed similar to microarray data. Compared with the control group, the cells overexpressing PPARG plus ROSI altered more genes compared with PPARG without ROSI. Among the genes involved in the upstream transcription factor regulation network, the expression level of* NR1H3*,* PPARG*,* SREBF2,* and* PPARD* by qPCR was similar to microarray, whereas data of* SREBF1* and* PPARGC1A* were less sensitive by microarray compared with qPCR (Figure 4). A contrasting response between microarray and qPCR was also observed for* FASN*.

### 3.5. Upstream Regulators

**Figure 5:**
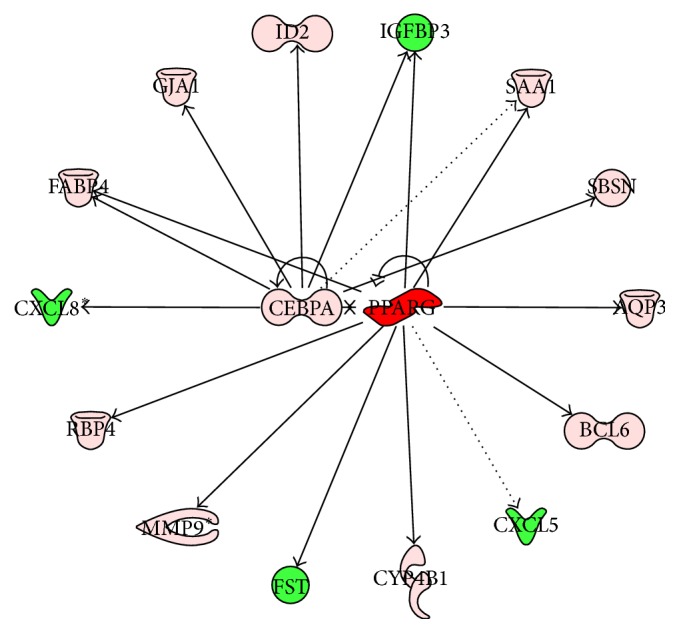
Ingenuity pathway upstream network analysis of differentially expressed genes (DEG) between cells treated with Ad-PPARG and those with Ad-GFP. Upstream regulators are located at the center of the network and downstream genes are located in the periphery. In the network, their downstream genes are also reported. Genes with red background are upregulated: red color (high upregulation) to light red color (moderate upregulation). Genes with green background were downregulated: green color (highly inhibited) to light red color (moderately highly inhibited). Arrows denote direct (solid lines) or indirect (dotted lines) interactions among genes.

**Figure 6:**
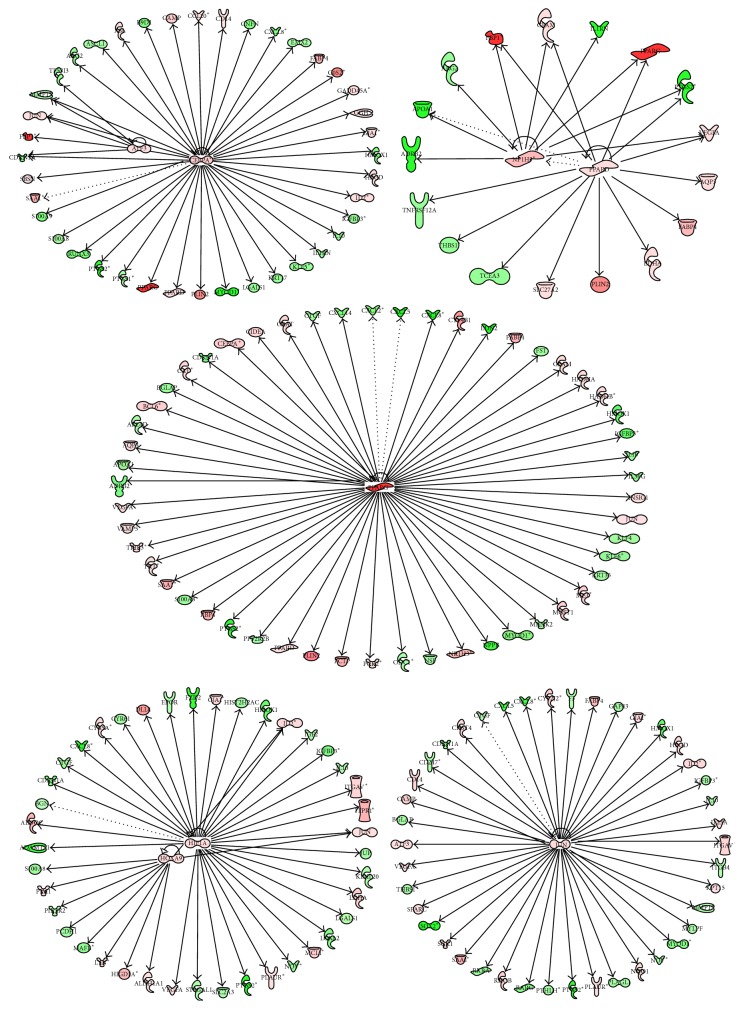
Ingenuity pathway upstream network analysis of differentially expressed genes (DEG) between cells treated with Ad-PPARG and rosiglitazone and those with Ad-GFP. Only upregulated transcription factors are shown in this network. Upstream regulators are located at the center of the network and downstream genes are located in the periphery. In the network, their downstream genes are also reported. The description of the color background and arrows in this figure is the same as Figure 5.

**Figure 7:**
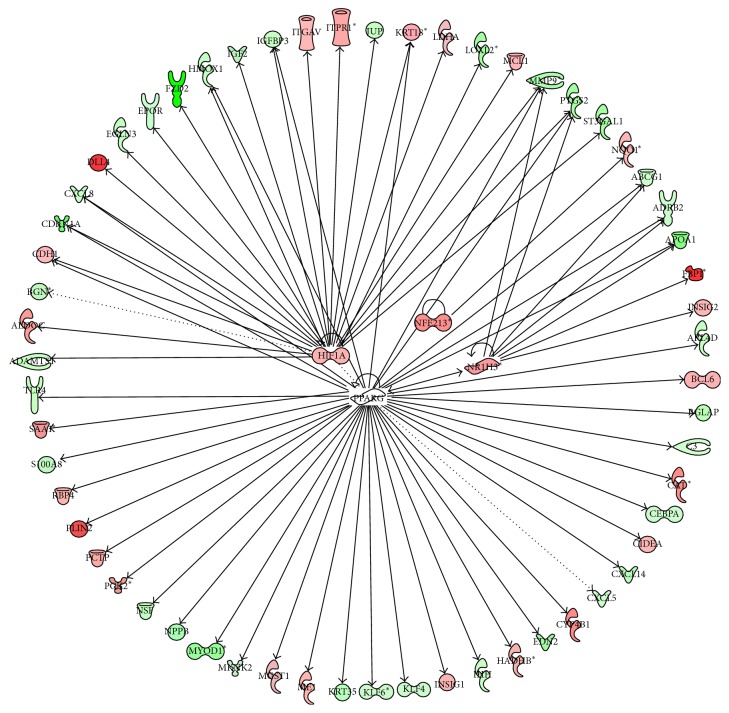
Ingenuity pathway upstream network analysis of differentially expressed genes (DEG) between cells treated with Ad-PPARG and rosiglitazone and those with Ad-PPARG. Only upregulated transcription factors are shown in this network. Upstream regulators are located at the center of the network and downstream genes are located in the periphery. In the network, their downstream genes are also reported. The description of the color background and arrows in this figure is the same as Figure 5.

Consistent with the number of DEG, there were a high number of upstream transcription regulators in the comparisons of PPARG+ROSI versus CON and PPARG+ROSI versus PPARG. All the upstream upregulated transcription regulators and their potential targets are depicted in Figures 5, 6, and 7. Among the upsteam transcription regulators with PPARG versus CON, there were two related to lipid metabolism including* PPARG* and CCAAT/enhancer binding protein (*C/EBP*), alpha (*CEBPA*). Comparing PPARG+ROSI with CON, eight upstream transcription regulators were upregulated: activating transcription factor 3 (*ATF3*),* CEBPA*, Jun protooncogene (*JUN*), homeobox A9 (*HOXA9*), hypoxia inducible factor 1, alpha* (HIF1A*), NR1H3, peroxisome proliferator-activated receptor delta (*PPARD*), and* PPARG* (Figure 6). Among them,* CEBPA*,* NR1H3*,* PPARD,* and* PPARG* are classical transcription factors related to lipid metabolism. Compared with PPARG+ROSI versus CON, the comparison of PPARG+ROSI with PPARG had a lower number of upregulated upstream transcription regulators including HIFA, nuclear factor, erythroid 2-like 3 (*NFE2L3*),* NR1H3,* and* PPARG* (Figure 7).

A few upstream transcription regulators were inhibited in the comparison of PPARG+ROSI versus CON and PPARG+ROSI versus PPARG (Figures S1 and S2). In comparison of PPARG+ROSI with CON, the transcription regulators early growth response 1 (*EGR1*), CXXC finger protein 1 (*CXXC1*), neurogenin 1 (*NEUROG1*), Protein Inhibitor of Activated STAT, 1 (*PIAS1*), pleomorphic adenoma gene-like 1 (*PLAGL1*), Kruppel-Like factor 4 (*KLF4*),* RXRA*,* GFI1B*,* KLF5*,* KLF6*,* RARG*,* MYOD1*, and* SOX2* were inhibited. Similar to PPARG+ROSI versus CON, the genes expression of* NEUROD1*,* KLF4*,* KLF6*,* CXXC1*,* KLF5*,* RXRA*, myogenic differentiation 1 (*MYOD1*), PIAS1, sex determining region Y box 2 (SOX2), and growth factor independent 1B transcription repressor (GFI1B) was also inhibited in the comparison of PPARG+ROSI with PPARG.

## 4. Discussion

Due to the unavailability of goat microarrays and the fact that structural genome of goats is closely related to that of bovine species, bovine arrays have been successfully adapted and applied in studies with goat mammary tissue [20, 25], goat ovary [26], and goat milk leukocytes [27]. To further explore the transcriptome alteration by PPARG gain of function, a commercial whole-transcriptome bovine microarray was used in the present study. The data revealed close to 1,000 DEG altered by overexpression of PPARG plus the chemical agonist ROSI. The most impacted category by PPARG was related to metabolism, which agrees with the previous findings demonstrating that PPARG plays a central role in adipogenesis [28]. Furthermore, analysis of a subset of genes by qPCR revealed a high degree of agreement with microarray data.

The DIA is efficient for the analysis of data from multiple treatment comparisons [24, 29]. Among the overall most impacted pathways in present study, the “fatty acid elongation in mitochondria,” “glycosaminoglycan biosynthesis-keratan sulfate,” and “glycosphingolipid biosynthesis-globo series” are novel and of biological interest. In adipose cells,* PPARG* promotes the uptake of fatty acids and storage as energy [7]. Our previous data also revealed that* PPARG* stimulated the expression of genes related to triacylglycerol (TAG) synthesis in GMEC [3, 12]. Thus, we expected to find that TAG synthesis would be the most impacted pathway in the present study. The finding that “fatty acid elongation in mitochondria” was the most impacted is supported by the high expression of hydroxyacyl-CoA dehydrogenase, alpha subunit (*HADHA*), and hydroxyacyl-CoA dehydrogenase, beta subunit (*HADHB*), both of which are the rate-limiting enzymes for fatty acid elongation. Further, these genes appear to be potential PPARG target genes in GMEC. Consistent with promoting fatty acid elongation, the uptake of long-chain fatty acid was also induced because* CD36* [3] and solute carrier family 27 (fatty acid transporter), member 6 (*SLC27A6*), were upregulated (Figure 2).

Both qPCR and microarray revealed that the expression of long-chain acyl-CoA synthetase 1 (*ACSL1*) was enhanced by overexpression of PPARG with ROSI (Figure 2). ACSL1 catalyzes the conversion of free fatty acids (FFAs) into their activated acyl-CoA derivatives, which are in turn used in the cell for *β*-oxidation, synthesis, or reacylation of many different cellular lipids or other cellular processes. Previous data suggested that FA activation in bovine mammary tissue occurs primarily via ACSL1 due to the fact that its mRNA is the most predominant among ACSL isoforms [30, 31].

Synthesis of very-long-chain FA is carried out by fatty acid desaturases 1 (*FADS1*) and 2 (FADS2), which add double bonds at the Δ5 and Δ6 position of PUFA and synthesize eicosapentaenoic acid (20:5n-3) and docosahexaenoic acid (22:6n-3). In this study, the fact that the expression of* FADS1* was significantly upregulated in PPARG-overexpressing cells indicated that this nuclear receptor may enhance the biosynthesis of polyunsaturated fatty acids. These data suggested that* FADS1* may be a target of PPARG. Hence, we hypothesize that the increase of omega-3/omega-6 ratio in milk fat could be achieved through the activation of PPARG in mammary cells. In fact, the hypothesis is supported by the fact that the subcategory “biosynthesis of unsaturated fatty acid” was among the top 30 categories in this study (File S3).

The perilipin (PAT) family [32–34] and cell death-inducing DFF45 like effector (*CIDE*) family [35, 36] play a pivotal role in lipid formation. In the present study, the marked upregulation of* PLIN2* in PPARG-overexpressing GMEC is consistent with recent data indicating that* PPARG* could directly bind to the promoter of PLIN2 and modulate the lipid formation in GMEC [13]. Less is known about the role of* CIDEA* in lipid droplet formation in ruminant mammary cells; however, it was the only CIDE isoform which was upregulated significantly after overexpression of PPARG plus ROSI. This indicates that* CIDEA* is a target of PPARG.

In addition to the PPAR family, the key transcription factors* SREBF1*,* NR1H3*,* CEBPA*,* H1F1A*,* JUN,* and* HOXA9* also had a significant change in response to the* PPARG* gain of function with or without ROSI. The cross talk between PPARG and* SREBF1* and* NR1H3* was described in our recent papers [2, 3, 12] and completely agrees with the present data that expression of* SREBF1* and* NR1H3* was enhanced by the overexpression of* PPARG* plus ROSI. The data from the IPA analysis indicating that overexpression of PPARG down- or upregulated these upstream transcription factors further supports our previous hypothesis that* PPARG* regulates the gene network related to fatty acid metabolism in a direct or indirect manner [3, 12]. Overall, the results indicated that goat mammary tissue relies heavily on PPARG regulation of genes to induce copious milk fat synthesis and secretion.

The pathway “glycosaminoglycan biosynthesis-keratan sulfate” is involved in the synthesis of keratan sulfate (KS); thus, its marked activation indicated that* PPARG* could control inflammatory response via regulating the synthesis of keratan sulfate. The hypothesis is consistent with the role of* PPARG* in inflammation in nonruminants [37–40]. Thus, this finding is novel and more research on KS synthesis seems warranted to better understand its role in the process inflammation, for example, during onset of mastitis.

The high activation of “pentose phosphate pathway” after overexpression of* PPARG* suggests that it promoted the efficient utilization of glucose in GMEC to generate substrates supporting other cellular processes. The high activation of glucose oxidation or other carbohydrate metabolism pathways in PPARG-overexpressing GMEC supports the view of a mechanism whereby* PPARG* alters metabolic pathways in lactating mammary gland; that is,* PPARG* promotes carbohydrate metabolism to produce intermediates to serve other aspects of milk fatty acid metabolism and lactose synthesis [41].

Due to the limitation of the microarray platform used [29], the interpretation of the findings from the present study has some limitations. For instance, the microarray platform used could not completely cover the genes with functional annotation in the goat genome. In addition, the difference between the goat and bovine genome will unavoidably miss some genes. In the future, goat specific oligo microarrays or next-generation sequencing should be used to confirm the transcriptome alterations caused by the* PPARG* gain of function. In that context, however, the present transcriptome analysis provides an initial global insight into the biological processes altered by* PPARG* in ruminant mammary cells.

## 5. Conclusions

Using cross-species hybridization microarray data, the present data support a role for* PPARG* activation on biological processes including and going beyond milk fat synthesis. The data indicated an overall increase in metabolism with large increase in anabolism, particularly involving fatty acid synthesis and glucose utilization. Most impacted terms underscored the regulatory role of* PPARG* in fatty acid elongation. The fact that pentose phosphate pathway was highly activated by* PPARG* suggests an important role in carbohydrate metabolism to produce intermediates for milk fatty metabolism.

The upstream regulator analysis indicated that* PPARG* controls molecular processes through an extensive level of cross talk with other signaling pathways, for example,* JUN* and* CEBPA*. All these data support our previous hypothesis that* PPARG* plays a central role in milk fatty metabolism in GMEC. In addition, the data also uncovered a likely role of* PPARG* in the GMEC response to inflammation via the “glycosaminoglycan biosynthesis-keratan sulfate” pathway. In conclusion, the data highlighted a strong transcriptional regulation of* PPARG* in the metabolism in GMEC.

## Supplementary Material

Selected gene names and primers used in this study.

## References

[1] Haenlein G. F. W. (2004). Goat milk in human nutrition. *Small Ruminant Research*.

[2] Shi H. B., Luo J., Yao D. W. (2013). Peroxisome proliferator-activated receptor-*γ* stimulates the synthesis of monounsaturated fatty acids in dairy goat mammary epithelial cells via the control of stearoyl-coenzyme A desaturase. *Journal of Dairy Science*.

[3] Shi H. B., Zhao W. S., Luo J. (2014). Peroxisome proliferator-activated receptor *γ*1 and *γ*2 isoforms alter lipogenic gene networks in goat mammary epithelial cells to different extents. *Journal of Dairy Science*.

[4] Kawai M., Rosen C. J. (2010). PPAR*γ*: a circadian transcription factor in adipogenesis and osteogenesis. *Nature Reviews Endocrinology*.

[5] Tontonoz P., Spiegelman B. M. (2008). Fat and beyond: the diverse biology of PPAR*γ*. *Annual Review of Biochemistry*.

[6] Heikkinen S., Auwerx J., Argmann C. A. (2007). PPAR*γ* in human and mouse physiology. *Biochimica et Biophysica Acta (BBA)—Molecular and Cell Biology of Lipids*.

[7] Lehrke M., Lazar M. A. (2005). The many faces of PPAR*γ*. *Cell*.

[8] Lee G., Elwood F., McNally J. (2002). T0070907, a selective ligand for peroxisome proliferator-activated receptor *γ*, functions as an antagonist of biochemical and cellular activities. *Journal of Biological Chemistry*.

[9] Zou R., Xu G., Liu X.-C. (2010). PPAR*γ* agonists inhibit TGF-*β*-PKA signaling in glomerulosclerosis. *Acta Pharmacologica Sinica*.

[10] Glas J., Seiderer J., Markus C. (2011). Role of PPARG gene variants in inflammatory bowel disease. *Inflammatory Bowel Diseases*.

[11] Kadegowda A. K. G., Bionaz M., Piperova L. S., Erdman R. A., Loor J. J. (2009). Peroxisome proliferator-activated receptor-*γ* activation and long-chain fatty acids alter lipogenic gene networks in bovine mammary epithelial cells to various extents. *Journal of Dairy Science*.

[12] Shi H. B., Luo J., Zhu J. J. (2013). PPAR*γ* regulates genes involved in triacylglycerol synthesis and secretion in mammary gland epithelial cells of dairy goats. *PPAR Research*.

[13] Kang Y., Hengbo S., Jun L. (2015). PPARG modulated lipid accumulation in dairy GMEC via regulation of ADRP gene. *Journal of cellular biochemistry*.

[14] Shahzad K., Bionaz M., Trevisi E., Bertoni G., Rodriguez-Zas S. L., Loor J. J. (2014). Integrative analyses of hepatic differentially expressed genes and blood biomarkers during the peripartal period between dairy cows overfed or restricted-fed energy prepartum. *PLoS ONE*.

[15] Moisá S. J., Shike D. W., Graugnard D. E. (2013). Bioinformatics analysis of transcriptome dynamics during growth in Angus cattle longissimus muscle. *Bioinformatics and Biology Insights*.

[16] Loor J. J., Everts R. E., Bionaz M. (2007). Nutrition-induced ketosis alters metabolic and signaling gene networks in liver of periparturient dairy cows. *Physiological Genomics*.

[17] Schibler L., Vaiman D., Oustry A., Giraud-Delville C., Cribiu E. P. (1998). Comparative gene mapping: a fine-scale survey of chromosome rearrangements between ruminants and humans. *Genome Research*.

[18] Ollier S., Robert-Granié C., Bernard L., Chilliard Y., Leroux C. (2007). Mammary transcriptome analysis of food-deprived lactating goats highlights genes involved in milk secretion and programmed cell death. *The Journal of Nutrition*.

[19] Pisoni G., Castiglioni B., Stella A. (2008). Microarray analysis of gene expression of milk leukocytes in healthy goats. *Veterinary Research Communications*.

[20] Ollier S., Leroux C., de la Foye A., Bernard L., Rouel J., Chilliard Y. (2009). Whole intact rapeseeds or sunflower oil in high-forage or high-concentrate diets affects milk yield, milk composition, and mammary gene expression profile in goats. *Journal of Dairy Science*.

[21] Wang Z., Luo J., Wang W., Zhao W., Lin X. (2010). Characterization and culture of isolated primary dairy goat mammary gland epithelial cells. *Chinese Journal of Biotechnology*.

[22] Lin X.-Z., Luo J., Zhang L.-P., Wang W., Shi H.-B., Zhu J.-J. (2013). MiR-27a suppresses triglyceride accumulation and affects gene mRNA expression associated with fat metabolism in dairy goat mammary gland epithelial cells. *Gene*.

[23] Akbar H., Cardoso F. C., Meier S. (2014). Postpartal subclinical endometritis alters transcriptome profiles in liver and adipose tissue of dairy cows. *Bioinformatics and Biology Insights*.

[24] Bionaz M., Periasamy K., Rodriguez-Zas S. L., Hurley W. L., Loor J. J. (2012). A novel dynamic impact approach (DIA) for functional analysis of time-course omics studies: validation using the bovine mammary transcriptome. *PLoS ONE*.

[25] Faucon F., Rebours E., Bevilacqua C. (2009). Terminal differentiation of goat mammary tissue during pregnancy requires the expression of genes involved in immune functions. *Physiological Genomics*.

[26] Magalhães-Padilha D. M., Geisler-Lee J., Wischral A. (2013). Gene expression during early folliculogenesis in goats using microarray analysis. *Biology of Reproduction*.

[27] Pisoni G., Moroni P., Genini S. (2010). Differentially expressed genes associated with *Staphylococcus aureus* mastitis in dairy goats. *Veterinary Immunology and Immunopathology*.

[28] Lowell B. B. (1999). PPAR*γ*: an essential regulator of adipogenesis and modulator of fat cell function. *Cell*.

[29] Bionaz M., Periasamy K., Rodriguez-Zas S. L. (2012). Old and new stories: revelations from functional analysis of the bovine mammary transcriptome during the lactation cycle. *PLoS ONE*.

[30] Mashek D. G., Coleman R. A. (2006). Cellular fatty acid uptake: the contribution of metabolism. *Current Opinion in Lipidology*.

[31] Bionaz M., Loor J. J. (2008). Gene networks driving bovine milk fat synthesis during the lactation cycle. *BMC Genomics*.

[32] Chong B. M., Reigan P., Mayle-Combs K. D., Orlicky D. J., McManaman J. L. (2011). Determinants of adipophilin function in milk lipid formation and secretion. *Trends in Endocrinology and Metabolism*.

[33] Bickel P. E., Tansey J. T., Welte M. A. (2009). PAT proteins, an ancient family of lipid droplet proteins that regulate cellular lipid stores. *Biochimica et Biophysica Acta—Molecular and Cell Biology of Lipids*.

[34] Wilfling F., Haas J. T., Walther T. C., Farese R. V. (2014). Lipid droplet biogenesis. *Current Opinion in Cell Biology*.

[35] Yang H., Galea A., Sytnyk V., Crossley M. (2012). Controlling the size of lipid droplets: lipid and protein factors. *Current Opinion in Cell Biology*.

[36] Singaravelu R., Lyn R. K., Srinivasan P. (2013). Human serum activates CIDEB-mediated lipid droplet enlargement in hepatoma cells. *Biochemical and Biophysical Research Communications*.

[37] Wan Y., Saghatelian A., Chong L.-W., Zhang C.-L., Cravatt B. F., Evans R. M. (2007). Maternal PPAR*γ* protects nursing neonates by suppressing the production of inflammatory milk. *Genes & Development*.

[38] Genini S., Badaoui B., Sclep G. (2011). Strengthening insights into host responses to mastitis infection in ruminants by combining heterogeneous microarray data sources. *BMC Genomics*.

[39] Ji P., Drackley J. K., Khan M. J., Loor J. J. (2014). Inflammation- and lipid metabolism-related gene network expression in visceral and subcutaneous adipose depots of Holstein cows. *Journal of Dairy Science*.

[40] Masoodi M., Kuda O., Rossmeisl M., Flachs P., Kopecky J. (2015). Lipid signaling in adipose tissue: connecting inflammation & metabolism. *Biochimica et Biophysica Acta—Molecular and Cell Biology of Lipids*.

[41] Bionaz M., Chen S., Khan M. J., Loor J. J. (2013). Functional role of PPARs in ruminants: potential targets for fine-tuning metabolism during growth and lactation. *PPAR Research*.

